# Hearing ability of prairie voles (*Microtus ochrogaster*)

**DOI:** 10.1121/10.0024357

**Published:** 2024-01-23

**Authors:** Emily M. New, Jessica A. Hurd, Genesis A. Alarcon, Cameron S. Miller, Peyton A. Williams, Nathaniel T. Greene, Casey E. Sergott, Ben-Zheng Li, Tim C. Lei, Elizabeth A. McCullagh

**Affiliations:** 1 Department of Integrative Biology, Oklahoma State University, Stillwater, Oklahoma 74078, USA; 2 Department of Otolaryngology – Head and Neck Surgery, University of Colorado School of Medicine, Aurora, Colorado 80045, USA; 3 Department of Electrical Engineering, University of Colorado Denver, Denver, Colorado 80204, USA; 4 Department of Physiology and Biophysics, University of Colorado Anschutz Medical Campus, Aurora, Colorado 80045, USA

## Abstract

The hearing abilities of mammals are impacted by factors such as social cues, habitat, and physical characteristics. Despite being used commonly to study social behaviors, hearing of the monogamous prairie vole (*Microtus ochrogaster*) has never been characterized. In this study, anatomical features are measured and auditory brainstem responses (ABRs) are used to measure auditory capabilities of prairie voles, characterizing monaural and binaural hearing and hearing range. Sexually naive male and female voles were measured to characterize differences due to sex. It was found that prairie voles show a hearing range with greatest sensitivity between 8 and 32 kHz, binaural hearing across interaural time difference ranges appropriate for their head sizes. No differences are shown between the sexes in binaural hearing or hearing range (except at 1 kHz), however, female voles have increased amplitude of peripheral ABR waves I and II and longer latency of waves III and IV compared to males. The results confirm that prairie voles have a broad hearing range, binaural hearing consistent with rodents of similar size, and differences in amplitudes and thresholds of monaural physiological measures between the sexes. These data further highlight the necessity to understand sex-specific differences in neural processing that may underly variability in responses between sexes.

## I. INTRODUCTION

The ability to localize sound sources is a critical task for animals and aids in the ability to forage for food, communicate with conspecifics, mate, and avoid predators, among many tasks. In mammals, estimation of spatial locations of sound sources can be approximated using interaural time difference (ITD) and interaural level difference (ILD) information between the two ears due to head size and pinna morphology.^1^ Processing of these cues occurs in the auditory brainstem in discrete areas that integrate ITD and ILD information from each ear. Which cues are available to different species and their hearing ranges are important to better understand the variability of the auditory system across taxa.

Prairie voles (*Microtus ochrogaster*) are a species of burrowing, grassland-dwelling rodent which possess important traits that may impact their sound localization ability and hearing. They are socially monogamous, exhibiting biparental care, where both sexes are highly responsive to social and parental cues such as ultrasonic pup vocalizations.^2,3^ Prairie vole colonies are typically composed primarily of extended families in which alloparenting is frequently observed, including behavioral and physiological responsiveness to pup vocalizations.^4–6^ They occupy subterranean and terrestrial environments. Many rodents with exclusive subterranean habitats have limited or reduced acoustic spatial ability.^7^ Middle ear structures would indicate that prairie voles should have a best hearing range of between 8 and 16 kHz with cutoff of around 50–60 kHz (based on data from a closely related species, *Microtus arvalis*^8^). However, most studies on prairie vole hearing have focused on their vocalizations and social behavior and not on their actual reception of sound and spatial hearing.^9^

Less than 1% of published auditory studies in rodents controlled for sex differences and when data compared sexes, explicitly 40 out of 69 studies (∼58%) found significant differences between sexes.^10^ Often the consideration of social structure or parental care of species studied is not specifically considered as a factor that might influence hearing, particularly influencing the reception of sound. In most rodents, much of the parental care falls on the mother. Although paternal care is widely associated with social monogamy, only 59% of socially monogamous mammal species exhibit paternal care of young. Approximately 6% of rodent species are socially monogamous,^11^ including the prairie vole,^12^ California mouse (*Peromyscus californicus*), and Mongolian gerbil (*Meriones unguiculatus*).^10^ All three of these species also engage in paternal care of young,^12^ although prairie voles and California mice exhibit a more equal distribution of parental workload than Mongolian gerbils.^13–15^ Whereas the parents of some social rodent species will communally raise young, naive male prairie voles often engage in a level of alloparental care that is remarkable and uncharacteristic of nearly all virgin male rodents.^12^

In several species of laboratory mice and rats, numerous sex differences in various aspects of the auditory system are documented.^10^ Interestingly, significantly fewer consistent sex differences have been established in the auditory function of monogamous rodent models, such as California mice, which exhibit no auditory sex differences,^16^ and Mongolian gerbils, where sex-based auditory differences are only observed with regard to age-related degeneration.^10^ Additionally, there are conflicting reports as to the presence of auditory sex differences in guinea pigs, which exhibit variation in the presence or absence of social monogamy and/or parental care.^10,17^ This suggests that it is possible that sex-based auditory differences might be related to the degree of difference in parental care by the sexes. Given the high degree of parental and alloparental care exhibited by male prairie voles, they may serve as an ideal species to further examine the possibility of this relationship.

In this study, we report the hearing ability of the monogamous prairie vole using auditory brainstem responses (ABRs) to measure binaural and monaural hearing in this species and between the sexes. We also measured head size, pinna morphology, and head-related transfer functions (HRTFs), which are additional contributors to horizontal and vertical sound localization^18^ (although horizontal features are studied specifically here). We predict that as a result of their biparental care and monogamous behaviors, there would be no differences in hearing between the sexes, and prairie voles would have hearing ability consistent with middle ear anatomical predictions independent of sex.

## II. MATERIALS AND METHODS

All experiments complied with all applicable laws, National Institutes of Health (NIH) guidelines, and were approved by the Oklahoma State University Institutional Animal Care and Use Committee (IACUC).

### A. Subjects

**FIG. 1.**
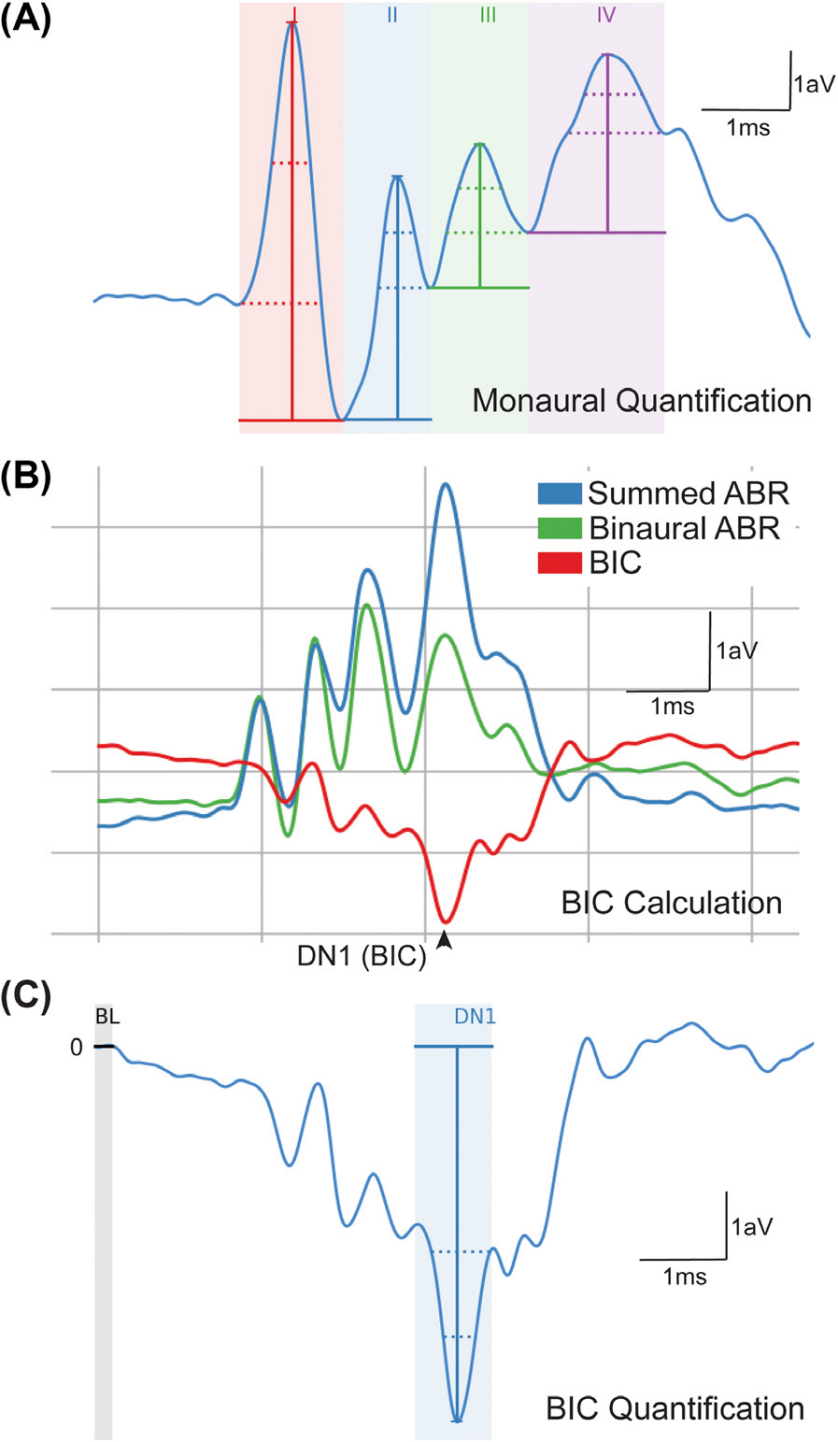
(Color online) Quantification of monaural/BIC ABRs. Monaural ABRs were quantified as peak to trough (absolute lowest point) for each wave (I, II, III, and IV) as shown in (A). The BIC was calculated as the binaural ABR subtracted from the sum of the two monaural ABRs at wave IV (B). The BIC amplitude (or DN1) was calculated relative to zero in the trace (BL or baseline), which was normalized across the entire recording (C). Scale is represented as one arbitrary voltage unit (aV) and ms as indicated.

**FIG. 2.**
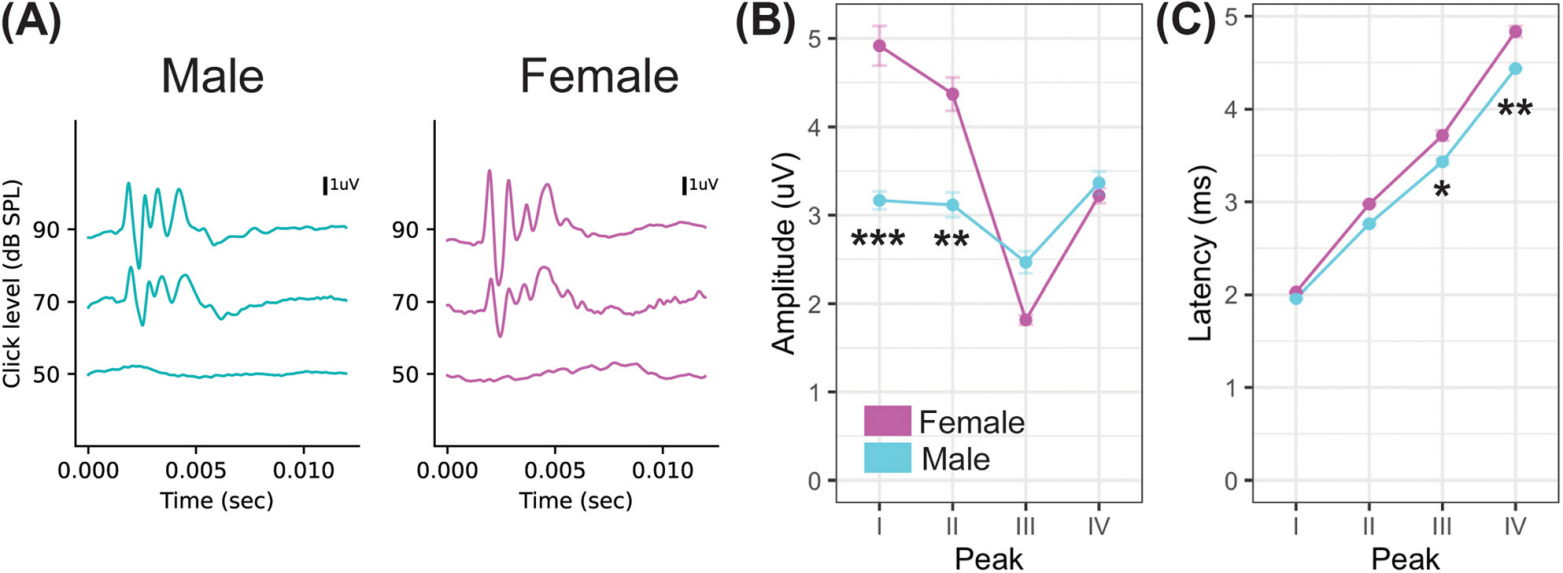
(Color online) Monaural ABRs were measured for each of the four peaks of the prairie vole ABR. (A) shows representative ABR traces to click stimuli for a representative male (left) and female (right) animal. The average responses for all male (cyan) and female (magenta) prairie voles for amplitude (B) and latency (C) across peaks I–IV generated by clicks at 90 dB SPL. Data represent ten males and nine females; * = *p* < 0.05, ** = *p* < 0.01, *** = *p* < 0.001.

**FIG. 3.**
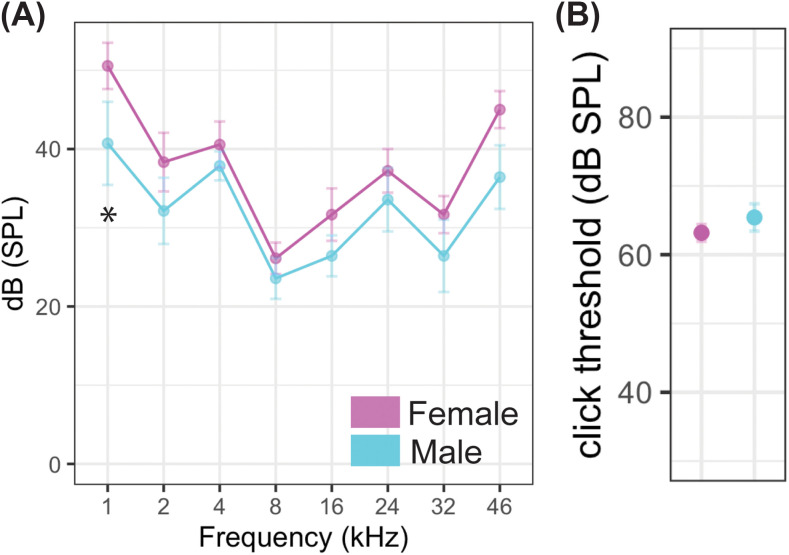
(Color online) Audiogram. Hearing threshold was measured across frequencies for male and female prairie voles (A). Mean responses for the sexes (magenta, female; cyan, male) show no differences between the sexes except at 1 kHz (*, *p* < 0.05). Click threshold for male and female voles, no significant difference. *N* is indicated in Table I.

**FIG. 4.**
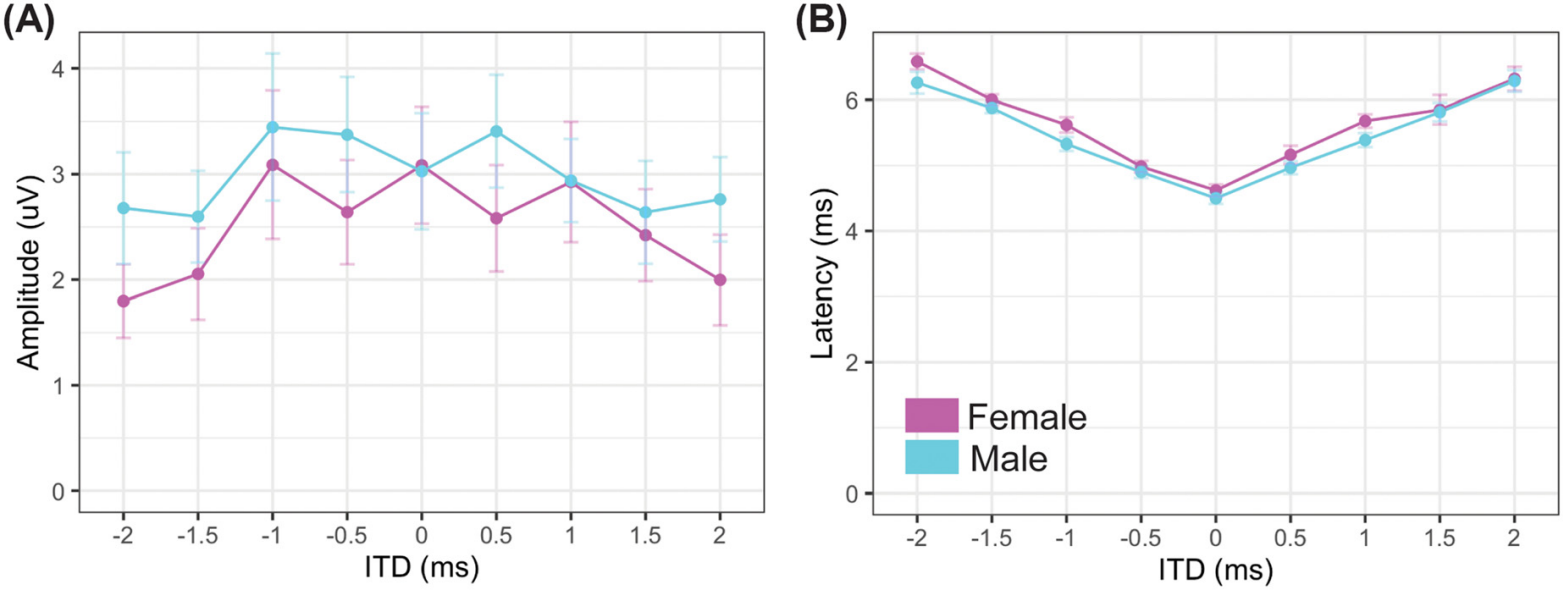
(Color online) BIC × ITD. The BIC as it varied with ITD was measured for both sexes. Mean responses for the sexes (magenta, female; cyan, male) show no differences between the sexes for either amplitude (C) or latency (D). *N* = 9 males and nine females.

**FIG. 5.**
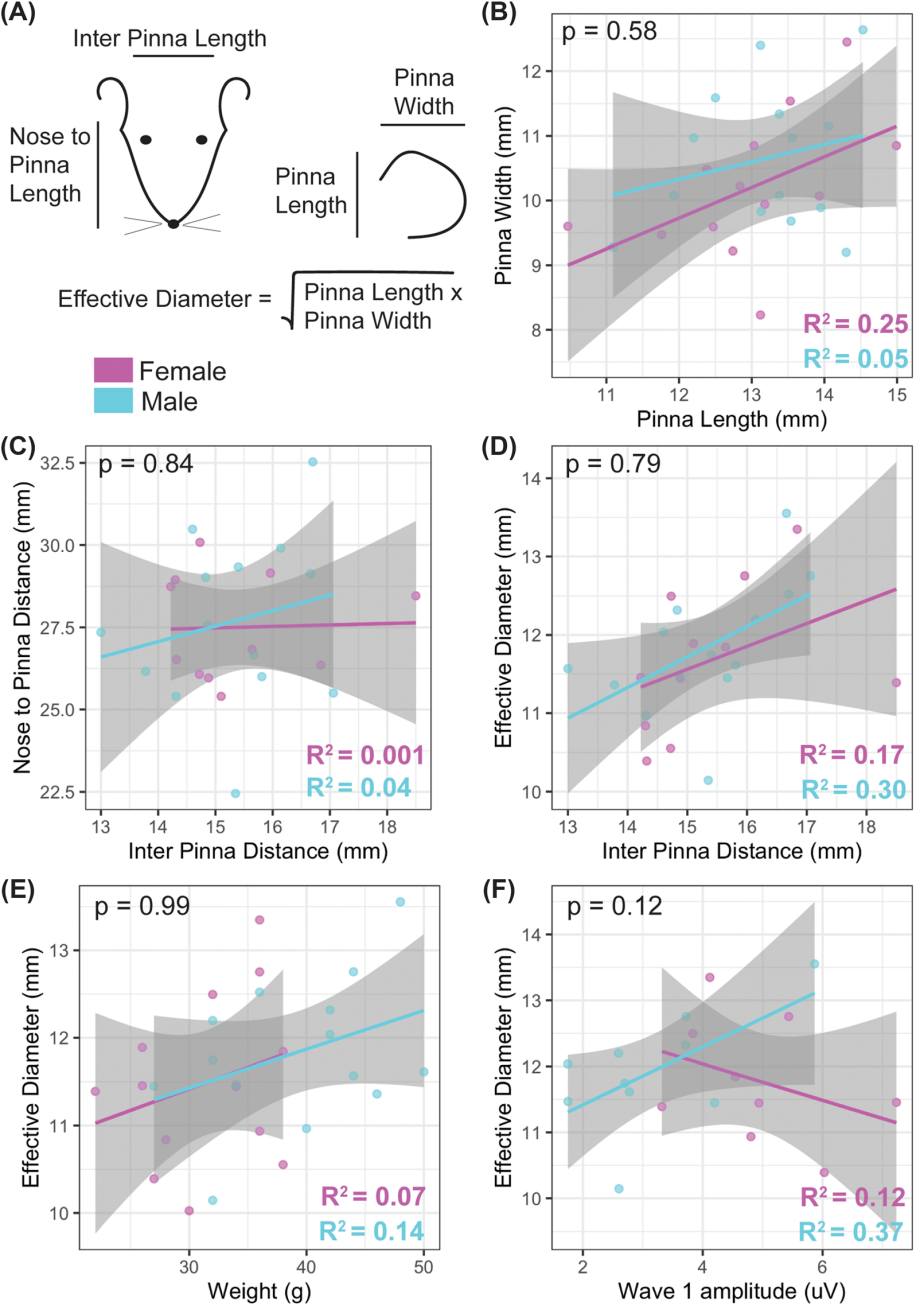
(Color online) Morphological features. Measurements of anatomical features of the head were taken for male and female prairie voles (A). Pinna size (B), head features (C), pinna diameter and inter pinna length (D), weight and pinna diameter I, and pinna diameter and wave I of the ABR (F) were compared (magenta, females; cyan, males; gray is standard error). Each dot represented data from one individual, *R*^2^ values are shown for individual regression analysis. *p*-values indicate ANOVA results comparing models with dependent variables and additional sex interaction model.

**FIG. 6.**
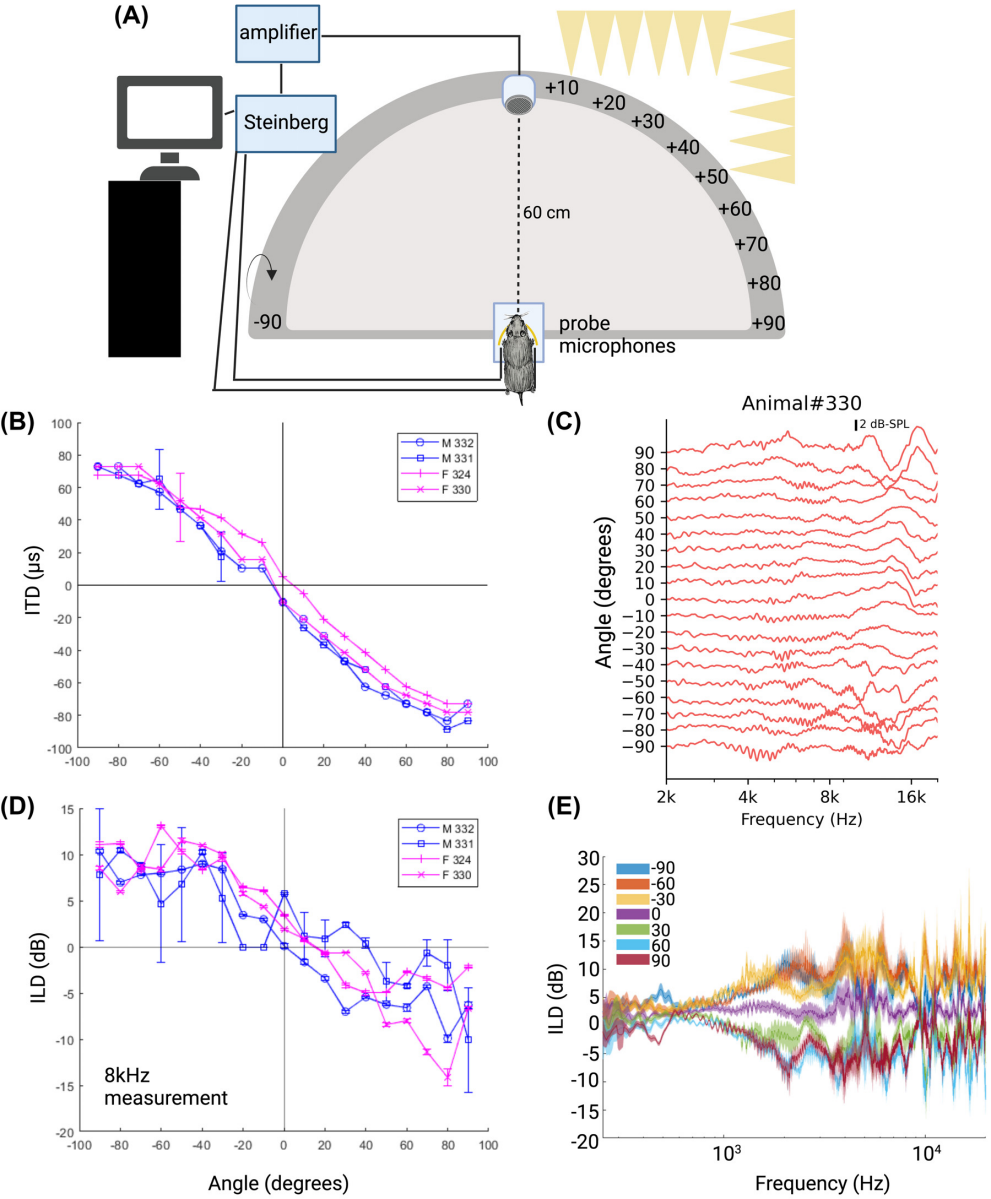
(Color online) HRTF measurements (A), depiction of the custom HRTF set up with movable speaker (center, 0°) that can move in 10° increments on the horizontal and vertical axes, allowing for 361 total measurements. For the current setup, measurements were only made in the 19 locations along the front-oriented horizontal axis as depicted. (B) shows the ITD as a function of angle for the two male (blue) and two female (magenta) animals. (C) shows an exemplar DTF from a male animal with spectral notches around 16 kHz. (D) shows an exemplar ILD as a function of angle of measurement at 8 kHz for the two male (blue) and two female (magenta) animals measured. (E) shows ILD as it varies with angle (colors depicted) and frequency for all animals. Figure was created with an online program (Ref. 77).

All experiments were conducted on laboratory-reared prairie voles (*M. ochrogaster*) obtained in 2020 from Dr. Tom Curtis's colony at the Oklahoma State Health Sciences Campus. Male and female animals were used (*N* for each experiment listed in the legends of Figs. 1–6 or Table I). Animals were between 180.6 ± 25.5 days old for females and 177.3 ± 27.0 days old for males (not significantly different *p* = 0.9309). Animals were housed with 1–2 individuals of similar age and sex after weaning and maintained on a 14:10 [light (6 a.m.):dark (8 p.m.) cycle]. All animals were sexually naive. Female prairie voles are induced ovulators, hence, there should be no impact of estrus stage on sex differences observed.^19^ Animals were tested between 9 a.m. and 3 p.m. during their light cycle.

**TABLE I.** Statistical measures for reported experiments [mean ± standard error (range, **N**)], test statistic, and *p*-values.

	Male	Female	Statistic	*p-*value
Peak amplitude (μV)			*t-*ratio	
I	3.2 ± 0.10	4.9 ± 0.22	3.733	*p* = 0.0004***
	(1.8–5.9, **10**)	(3.3–7.2, **9**)		
II	3.1 ± 0.14	4.2 ± 0.20	2.729	*p* = 0.008**
	(2.0–4.8, **10**)	(2.3–5.9, **9**)		
III	2.5 ± 0.13	1.8 ± 0.054	−1.384	*p* = 0.17
	(0.47–4.3, **10**)	(0.57–4.0, **9**)		
IV	3.4 ± 0.13	3.2 ± 0.085	−0.311	*p* = 0.76
	(1.2–4.9, **10**)	(2.1–4.6, **9**)		
Peak latency (ms)			*t*-ratio	
I	1.96 ± 0.03	2.02 ± 0.05	0.522	*p* = 0.61
	(1.8–2.2, **10**)	(1.7–2.3, **9**)		
II	2.76 ± 0.05	2.98 ± 0.06	1.635	*p* = 0.11
	(2.6–3.2, **10**)	(2.7–3.5, **9**)		
III	3.43 ± 0.06	3.71 ± 0.09	2.227	*p* = 0.03*
	(3.2–4.0, **10**)	(3.3–4.4, **9**)		
IV	4.43 ± 0.07	4.83 ± 0.12	3.137	*p* = 0.004**
	(4.1–5.1, **10**)	(4.3–5.9, **9**)		
Click threshold (dB SPL)			*t*-value	
	65.4 ± 1.99	63.2 ± 1.21	−0.957	*p* = 0.35
	(55–75,**12**)	(55–65,**11**)		
Frequency threshold (dB SPL)			*t*-ratio	
1 kHz	46.2 ± 7.2	50.6 ± 2.9	2.112	*p* = 0.04*
	(25–65, **7**)	(35–65, **9**)		
2 kHz	37.5 ± 6.4	38.3 ± 3.7	1.329	*p* = 0.19
	(25–55, **7**)	(25–65, **9**)		
4 kHz	40.0 ± 2.7	40.6 ± 2.9	0.579	*p* = 0.56
	(35–45, **7**)	(25–55, **9**)		
8 kHz	25.0 ± 2.7	26.1 ± 2.0	0.545	*p* = 0.59
	(15–35, **7**)	(15–35, **9**)		
16 kHz	28.8 ± 3.2	31.7 ± 3.3	1.124	*p* = 0.27
	(15–35, **7**)	(15–45, **9**)		
24 kHz	35.0 ± 3.8	37.3 ± 2.8	0.784	*p* = 0.44
	(15–45, **7**)	(25–45, **9**)		
32 kHz	28.8 ± 4.6	31.7 ± 2.4	1.124	*p* = 0.27
	(15–45, **7**)	(25–45, **9**)		
46 kHz	38.8 ± 4.2	45.0 ± 2.4	1.840	*p* = 0.072
	(25–55, **7**)	(35–55, **9**)		
BIC amplitude (μV)			*t*-ratio	
−2.0 ms	2.7 ± 0.53	1.8 ± 0.35	−1.227	*p* = 0.23
	(0.51–4.9, **9**)	(0.74–4.0, **9**)		
−1.5 ms	2.6 ± 0.44	2.1 ± 0.43	−0.757	*p* = 0.45
	(0.52–3.7, **9**)	(0.36–4.3, **9**)		
−1.0 ms	3.4 ± 0.70	3,1 ± 0.70	−0.498	*p* = 0.62
	(0.72–7.4, **9**)	(0.41–6.8, **9**)		
−0.5 ms	3.4 ± 0.55	2.6 ± 0.49	−0.841	*p* = 0.40
	(0.84–5.3, **9**)	(1.3–5.8, **9**)		
0.0 ms	3.0 ± 0.55	3.1 ± 0.56	0.079	*p* = 0.94
	(1.2–5.4, **9**)	(1.8–6.1, **9**)		
0.5 ms	3.4 ± 0.53	2.6 ± 0.50	−0.964	*p* = 0.34
	(0.39–5.1, **9**)	(0.5–5.1, **9**)		
1.0 ms	2.9 ± 0.39	2.9 ± 0.57	−0.019	*p* = 0.98
	(0.74–4.6, **9**)	(1.2–5.6, **9**)		
1.5 ms	2.6 ± 0.49	2.4 ± 0.44	−0.303	*p* = 0.76
	(0.59–4.7, **9**)	(0.85–4.5, **9**)		
2.0 ms	2.8 ± 0.40 (0.98–4.6, **9**)	2.0 ± 0.43 (0.16–4.4, **9**)	−1.064	*p* = 0.29
BIC latency (ms)			*t*-ratio	
−2.0 ms	6.3 ± 0.17 (5.1–6.8, **9**)	6.6 ± 0.12 (6.2–7.3, **9**)	1.784	*p* = 0.08
−1.5 ms	5.9 ± 0.08	6.0 ± 0.08	0.678	*p* = 0.49
	(5.5–6.3, **9**)	(5.6–6.4, **9**)		
−1.0 ms	5.3 ± 0.11	5.6 ± 0.11	1,608	*p* = 0.11
	(4.8–5.9, **9**)	(5.2–6.3, **9**)		
−0.5 ms	4.9 ± 0.09	5.0 ± 0.09	0.660	*p* = 0.51
	(4.6–5.3, **9**)	(4.6–5.5, **9**)		
0.0 ms	4.5 ± 0.09	4.6 ± 0.09	0.666	*p* = 0.21
	(4.1–4.8, **9**)	(4.3–5.1, **9**)		
0.5 ms	5.0 ± 0.10	5.2 ± 0.14	1.274	*p* = 0.21
	(4.5–5.4, **9**)	(4.8–6.0, **9**)		
1.0 ms	5.4 ± 0.11	5.7 ± 0.10	1.608	*p* = 0.11
	(4.9–5.9, **9**)	(5.3–6.1, **9**)		
1.5 ms	5.8 ± 0.14	5.9 ± 0.22	0.201	*p* = 0.84
	(5.0–6.3, **9**)	(4.2–6.5, **9**)		
2.0 ms	6.3 ± 0.16	6.3 ± 0.18	0.176	*p* = 0.86
	(5.2–6.8, **9**)	(5.0–7.0, **9**)		
Morphological measures			*t*-value	
Nose to pinna (mm)	27.7 ± 0.7	27.4 ± 0.4	−0.31973	*p* = 0.75
	(22.4–32.5, **14**)	(25.4–30.1, **12**)		
Inter pinna (mm)	15.3 ± 0.3	15.4 ± 0.7	0.87445	*p* = 0.40
	(13.0–17.1, **14**)	(14.2–18.5, **11**)		
Pinna width (mm)	10.7 ± 0.3	10.2 ± 0.3	−1.0904	*p* = 0.29
	(9.2–12.6, **14**)	(8.2–12.5, **13**)		
Pinna length (mm)	13.2 ± 0.3	13.0 ± 0.3	−0.51444	*p* = 0.61
	(11.1–14.5, **14**)	(10.5–15.0, **13**)		
Effective diameter (mm)	11.8 ± 0.2	11.5 ± 0.3	−0.99192	*p* = 0.33
	(10.1–13.6, **14**)	(10.0–13.3, **13**)		
Weight (g)	39.2 ± 1.9	31.5 ± 1.5	−3.2467	*p* = 0.0034**
	(27–50, **14**)	(22–38, **13**)		

### B. ABR acquisition

ABR recordings were conducted using methods similar to those described in our previous works.^20–23^ Briefly, animals were anesthetized intraperitoneal (i.p.) with a mixture of ketamine-xylazine (initial, 60 mg/kg ketamine, 10 mg/kg xylazine; maintenance, 25 mg/kg ketamine, 12 mg/kg xylazine) and placed on a recirculating heating pad in a small sound attenuating chamber (Noise Barriers, Lake Forest, IL). Once the animals were unresponsive to a toe-pinch stimulus, subdermal needle electrodes were placed under the skin at the apex (between the ears), reference (nape), and ground (back leg); this montage helps generate a maximal binaural interaction component (BIC) recording.^20,21,24^ Evoked potentials from the electrodes were amplified by a Tucker-Davis Technologies (TDT, Alachua, FL) RA4LI head stage (20× amplification) and TDT RA16PA preamplifier. Potentials were further amplified using a TDT multi input–output (I/O) processor RZ5 and recorded using custom PYTHON software. Data were processed using a second order 50–3000 Hz filter and averaged across 500–1000 repetitions per condition with 12 ms of recording time. Sound stimuli were generated using a TDT RP2.1 real-time processor controlled by custom PYTHON code at a sampling rate of 97 656.25 Hz. Sounds were presented to the animal through TDT MF-1 multi-field speakers (1–24 kHz) or TDT EC-1 electrostatic speakers (32–46 kHz), coupled with custom ear bars fitted with Etymotic ER-7C probe microphones (Etymotic Research Inc., Elk Grove Village, IL) for in-ear calibration.^25,26^ Stimuli were calibrated by in-ear microphones for every animal prior to recordings. Sound stimuli included transient (0.1 ms square wave pulse) click stimuli with alternating polarity and tone bursts (2 ms ± 1 ms on/off ramp) of varying frequencies and intensities (1–46 kHz). For monoaural ABR wave amplitude and latency quantification, click stimuli were presented at 90 dB sound pressure level (SPL). Stimuli were presented every 30 ms with an interstimulus standard deviation of 5 ms.

### C. Monaural ABRs

Evoked potentials in response to single ear click stimulation were recorded for each ear independently and quantified as peak amplitude (value from peak to absolute trough) and latency (time to peak amplitude) across the four peaks of the ABR waveform at 90 dB SPL [Fig. 1(A)].^27,28^ Monaural peak amplitude and latency for the left and right ears were then averaged for each animal to obtain an average monaural peak amplitude and latency.^21^ Click threshold was determined by decreasing the level of the sound in 10 dB SPL steps until there was no longer an ABR response recorded [Fig. 2(A) for exemplar male and female traces]. Threshold was next estimated to be the difference between the last level that elicited a response and the next lowest stimulation (i.e., if 60 dB SPL elicited a response and 50 dB SPL did not, threshold would be estimated as 55 dB SPL).^29^

### D. Audiogram

Threshold of ABR was determined across frequencies (1, 2, 4, 8, 16, 24, 32, and 46 kHz) to generate an audiogram of prairie vole hearing ranges. Briefly, animals were presented with tone pips (2 ms duration and 1 ms on/off ramp for a total of 4 ms) at varying intensities in 10 dB steps across frequencies. When a response was no longer observed, threshold was determined to be the difference between the last response and the subsequent lack of response (see above for click threshold). Visual inspection has been shown to provide similar results for threshold detection compared to objective algorithms^30^ in other studies using non-*Mus* species,^16^ therefore, we used visual inspection to determine the threshold by at least two different trained observers for each measurement.

### E. BIC with ITD

In addition to monaural stimulation, animals were presented with transient click stimuli to both ears simultaneously or with an ITD (–2 to +2 ms in 0.5 ms steps) at 90 dB SPL. The two monaural responses were summed and subtracted from the binaural ABR response to generate the BIC [Fig. 1(B)].^20,21,24,31^ BIC was then measured across ITDs for amplitude and latency. The amplitude of the BIC was determined as the peak relative to zero and baseline of the overall trace, which was set to zero [Fig. 1(C)].

### F. ABR data analysis

ABR traces were analyzed using custom PYTHON software. ABR and BIC peaks were automatically detected and labeled by searching local extremes. The detected peaks were then inspected and manually adjusted if inaccurate, for example, if a wave was misclassified or counted twice. Additionally, if a peak was determined to be not present, it could be deselected and not quantified. Values for amplitude and latency were then saved as separate files and collated across animals. Only animals with detectable peaks during click stimulus initially and at the end of the experiment were included in the analysis.

### G. Morphological characteristics

Morphological features were determined for each animal using 6 in. stainless steel electronic vernier calipers (DIGI-Science Accumatic digital caliper, Gyros Precision Tools, Monsey, NY, USA). Pinnae are the first anatomical features to play a role in hearing of an animal with external ears.^32^ We measured pinna size (length and width), effective diameter,^33^ distance between the pinna (inter pinna distance), and distance from the tip of the nose to the midpoint between the pinna, between the sexes [Fig. 5(A)]. Last, animals were weighed using a digital scale.

### H. HRTFs

To measure how the heads and pinnae of different individuals affect sound propagation, we performed a series of HRTFs on four new animals (two males and two females). These animals were not included in other experiments and were similar in age and morphological attributes across the sexes (average data for females, 166.5 days old, 31.2 g, pinna width 9.2 mm, pinna length 12.0 mm, inter pinna distance 12.9 mm, nose to pinna 26.3 mm; males, 166 days old, 34.5 g, pinna width 9.7 mm, pinna length 12.9 mm, inter pinna distance 11.4 mm, nose to pinna 23.0 mm).

Measurements were conducted in an anechoic chamber (SPL –20 dB, full surround with acoustic wedges, including the floor) or a sound attenuating chamber (86 × 112 × 86 in.; O'Neill Engineered Systems, Hartland, WI) with a custom-made speaker system array (single movable speaker 60 cm from the base/animal; Dayton Audio ND65-4, Springboro, OH), capable of moving 180° around the subject. On the vertical plane, 0° elevation was defined as directly in front of the animal while 180° was defined as directly behind the animal. On the horizontal plane, 0° was defined as directly overhead the animal while –90° and +90° were defined as directly on the left and right sides, respectively. Each speaker position was 10° apart, making for a total of 19 possible locations on the horizontal axis and 19 possible locations on the vertical axis for a total of 361 potential measurements. For this study, we took measurements at each location on the horizontal plane while keeping the elevation at 0° for a total of 19 measurement locations in front of the animal (+90 to –90). Animals were first sacrificed with a lethal dose of pentobarbital (120 mg/kg) shortly before placement in the chamber. The animal was then placed in the middle of the setup on an acrylic stand covered in acoustic foam with its head positioned exactly at the midpoint of the array. The subject's nose rested approximately 3 mm from the end of the acrylic stand [Fig. 6(A)]. The animal was secured to the platform using tape to ensure minimal movement while the microphones were placed into the ears. Animals were placed in a prone position similar to a natural resting position that the animal would assume when alive. ER-7C probe tube microphones (Etymotic Research Inc., Elk Grove Village, IL) were placed into the ear canal by threading the tubing through a hollow 14 G needle inserted behind the pinna. Care was taken that the probe microphone tip did not touch the ear canal and was placed similarly for all animals. Measurements were taken in REW (Room EQ Wizard, version 5.20.13 Pro upgrade, Montrose, Angus, Scotland, UK), a room acoustics software. A Steinberg UR22C (Steinberg Media Technologies GmBH, Hamburg, Germany) was used as the audio interface along with a stereo amplifier (Sony STR-DH190, New York, NY) to drive the speaker. Microphone calibration files provided by Etymotic were uploaded into REW and used as a reference for the software in addition to calibrating the SPL meter (internal to REW) with a dB meter and sound card calibration to ensure that the input and output levels were within 6 dBfs (full scale) of each other. Stimuli were 128k point logarithmically swept sines (–15 dBfs) and presented from 250 to 20 000 Hz over 3 s with a sampling rate of 44.1 kHz and acoustic timing reference. Each sweep was performed five times at each location, microphone responses were recorded simultaneously in both ears, amplified and digitized with the Steinberg UR22C, and preanalyzed and displayed by the REW software. Calibration measurements at each 10° increment (–90 to +90, 0° vertical) were taken without an animal present but with microphones placed in a location similar to the position with the animal and subtracted from the measured responses to eliminate any loudspeaker effects.

Amplitude spectra were calculated using a 512-point fast Fourier transform (FFT) and filtered with a 1/48 octave filter by the REW software. Data were exported as .txt files and processed in PYTHON or MATLAB. HRTFs were calculated as the gain of SPL between the animal recording and microphone-only recording. The mean across all HRTF positions was subtracted from each position to calculate the directional transfer function (DTF).^33–35^ ILDs were calculated as the frequency dependent difference in gain between the right ear microphone relative to the left ear microphone at each speaker position (in dB), and ITDs were calculated as the shift in the impulse response in the right ear relative to the left ear as determined by a shift in the cross correlation relative to the autocorrelation function (in ms).

### I. Statistical analyses

Figures were generated in *R* (Ref. 36) using ggplot2 (Ref. 37) and represent mean and standard error in line plots. Data were analyzed using linear mixed effects models to account for repeat observations within one animal (lme4; Ref. 38) with sex and condition (ITD, frequency, and peak) as fixed effects and animal as a random effect. Estimated marginal means (emmeans^39^) were used for pairwise comparisons across peaks, frequencies, and ITDs between the sexes. To control for multiple comparisons, emmeans implements a Tukey method for contrasts. Linear regression best fit with standard errors are displayed for morphological variables of each sex independently (*r*-squared and *p*-values listed in Fig. 5). Linear regression models with sex as an interaction variable were performed for relationships between morphological features and/or ABR waveforms. Analyses of variance (ANOVAs) were then performed on linear models with and without sex as an interaction variable to determine whether the dependent morphological variables depended on the sex of the individual. Two-tailed *t*-tests were performed to determine if there were differences in age between the sexes and click thresholds. Outliers were tested for by examining the data and calculating if values fell within three standard deviations from the mean for that group; animals that had values that were considered outliers were removed from analysis. Where values are indicated as statistically significant between the two sexes, “*” indicated a *p*-value of < 0.05, ** = *p* < 0.01, and *** = *p* < 0.0001. Figures 2–5 were generated in R (Ref. 36) using ggplot2 (Ref. 37) and Fig. 6 generated with MATLAB (The Mathworks, Inc. Version 2023a, Natick, MA). Line plots represent mean and standard error.

## III. RESULTS

We measured the hearing ability of prairie voles using ABRs and measured anatomical features that contribute to sound reception. Specifically, ABR responses (amplitude and latency) to monaural click stimuli, ABR thresholds across frequencies, amplitude, and latency of the BIC with varying ITDs, and morphological features (pinna size head characteristics and HRTFs) were measured between the sexes (Table I).

### A. Monaural ABRs

First, we measured male and female prairie vole's responses to monaural transient click stimuli at 90 dB SPL (Fig. 2). Prairie voles showed a characteristic ABR similar to other small rodents with four prominent peaks [I–IV, Fig. 2(A)]. These peaks had similar latencies to other species, appearing within the first 6–8 ms after the click stimulation.^20^ There were significant sex differences in the monaural peak amplitude of wave I and wave II ABR responses in prairie voles (*p* < 0.001, wave I; *p* < 0.01, wave II). Peak amplitude was significantly larger in female prairie voles for waves I and II of the ABR compared to males [Fig. 2(A), representative responses for each sex; Fig. 2(B), mean responses]. Latency of peaks was significantly longer in females for waves III and IV of the ABR [Fig. 2(C); *p* < 0.05, wave III; *p* < 0.01, wave IV). The linear mixed models did not show an overall significant sex-specific effect of amplitude when considering all ABR waves together (*t*-value –1.7, *p* = 0.10), although there was a significant effect of sex on latency across the peaks (*t*-value –2.18, *p* = 0.04).

### B. Audiogram

Next, we wanted to determine sex differences in frequency responses. Males had significantly lower low frequency hearing thresholds at 1 kHz compared to females (*p* = 0.04); however, they had comparable hearing to females at every other frequency (Fig. 3). Additionally, there was no significant effect of sex across frequencies (*t*-value –1.623, *p* = 0.12), therefore, the differences obsered at 1 kHz are not conclusive. Prairie voles show a broad hearing range with best hearing between 8 and 32 kHz as for other rodents of similar size^10^ and consistent with pup calls that have vocalization fundamental frequencies between 22 and 50 kHz.^40^ There were no significant differences in overall click threshold [Fig. 3(B); *p* = 0.35].

### C. BIC × ITD

Binaural hearing ability of prairie voles was assessed using the BIC of the ABR as it varies with ITD. The BIC has been shown to be a predictive biomarker of binaural hearing ability in many species and is responsive to hearing impairment.^20,24^ Unlike monaural ABRs, there was no difference between the sexes in either amplitude [Fig. 4(A)] or latency [Fig. 4(B)] of the BIC as it varied with ITD, including no significant effect of sex on the BIC amplitude (*t*-value 0.799, *p* = 0.43) or latency (*t*-value –1.412, *p* = 0.18) across ITD. The BIC amplitude was largest and BIC latency shortest around zero ITD, which is consistent with other species.^20,21^ BIC latency increased with longer ITDs while amplitude decreased as ITD increased.^20,31^

### D. Morphology

We did not observe any statistically significant differences between the sexes for any of the morphological features of the head and pinna or comparisons to ABR waveforms except for a significant difference in weight between the sexes (Table I, Fig. 5) with males weighing significantly more than females (*p* < 0.003, Table I). Pinna length and width were positively correlated, indicating that ears grew larger symmetrically [Fig. 5(B)]. Similarly, inter pinna length and nose to pinna distance [Fig. 5(C)], effective diameter of the pinna and inter pinna distance [Fig. 5(D)], and weight and effective diameter [Fig. 5(E)] were positively correlated, indicating that these attributes generally varied with an overall increased size of the individual's heads. Interestingly, pinna size in female voles was negatively correlated with wave I of the ABR while males showed a positive correlation [Fig. 5(F)], although these results were not significantly different between the sexes (*p* = 0.12).

### E. HRTF

To test the relationship between anatomical morphological features and auditory cues available to prairie voles between the sexes, we measured the HRTF of four additional voles (two males and two females; Fig. 6). Bilateral DTFs revealed relatively flat gains across all spatial locations for low frequencies (≲8 kHz) and increasing variability at higher frequencies. Some animals showed evidence of a spectral notch in the front locations (±20°) above ∼16 kHz; more consistently, variability in gain was observed for more lateral positions [Fig. 6(C)]. The ILD was calculated as the difference in gain across the two ears and was highly consistent across animals: ILD was low (<5 dB) for low frequencies (<4 kHz) and rose to 15–20 dB for higher frequencies (∼8 kHz) at lateral positions (>70°), although the absolute ILD value varied considerably with frequency at these high frequencies [similar to the variability observed in other mammalian species;^24,48^ Figs. 6(D) and 6(E)]. ITDs were calculated as the shift in the impulse response measured in each ear (constructed using the swept sine method) as determined by the peak in the cross correlation between the two signals. ITD was more consistent than ILD and varied between ± 80 *μ*s at lateral locations [Fig. 6(B)]. This delay corresponds to an acoustic travel time of approximately 27 mm, which is consistent with the widths of the voles' heads plus pinna prominence. Consistent with our pinnae and head size measurements, we did not see significant differences in any HRTF measurements between male and female voles, including DTF gain or ILD and ITD magnitudes.

## IV. DISCUSSION

This is the first study to characterize the hearing ability of prairie voles, including monaural and binaural hearing, hearing range, and pinna morphology between the sexes. Whereas we did not find significant differences in hearing range or binaural hearing ability between the sexes, we did show significant differences in monaural amplitude and latency, indicating sex-specific changes in auditory processing in this species. Prairie voles are particularly interesting rodents due to their social behaviors, although, as yet, little is known about their sound reception ability.^5^ Hearing ability is an important aspect of social communication that has been understudied in this rodent group. Our hearing range findings are consistent with work on other vole species^8^ and proposed hearing ranges based on vocalizations.^3,9^ However, this is one of the few studies to show differences in amplitude and latency of monaural ABRs between the sexes, particularly in a rodent model that exhibits biparental and alloparental care.

Most of the work regarding sex differences in rodent ABRs shows that there is differential susceptibility to manipulations that affect hearing between the sexes (reviewed in Ref. 10). Additionally, sex differences have been observed in clinical models of hearing disorders in several rodent species or strains,^10^ often as changes in hearing threshold or range. In contrast, our study showed subtle or no differences in click threshold or hearing range between the sexes. We observe differences in monaural hearing but no change in binaural hearing (as measured by the BIC). Consistent with no changes in binaural hearing, the monaural sex differences that we observe are increased amplitude of waves I and II in females, which are not thought to be part of the binaural pathway.^42^ However, we do show increased latency of waves III and IV of the ABR in females, which are thought to represent binaural areas. Whereas we show the largest BIC amplitude and shortest latency at zero ITD, similar to other studies,^20,21^ our overall BIC ITD relationship appears broader,^20,31^ which could be the result of differences in quantification methods or other factors. Our data are consistent with BIC amplitude decreasing with larger ITDs as well as latency of the BIC increasing with ITD.^20,31^ It is important to consider individual head morphology when analyzing ABR results. Although only overall weight was statistically significant, females had statistically similar head and pinna size to males, thus, differences in head morphology cannot explain the differences observed in ABR waves I and II. Consistent with the similar head dimensions, HRTF measurements reveal comparable binaural cues available to males and females. Interestingly, increased latency of responses in females is opposite to sex differences in ABR latencies in humans, which are typically shorter in women,^43,44^ and this could be due to species-specific or hormonal differences between ages and mating status of individuals studied. Further study exploring sex differences in hearing after mate-pairing in this species would be particularly interesting to study the impact of hormonal changes related to pair-bonding on hearing.

The choice of rodent model for studying hearing is often limited to genetically tractable models, such as rats or mice, despite behavioral and hearing range differences to humans. However, in comparison to other neuroscience fields, auditory function has been measured in many different species, in particular, rodents.^45^ There is substantial need to control for sex differences in auditory research^10^ with special regard to the degree of paternal care and other factors exhibited in species diversity. Compared to other rodents, prairie voles are somewhat unique in their monogamous mating style and male parenting, even when compared to other monogamous rodents,^12^ although, of course, there is variability to their monogamy (social versus genetic) and parental behaviors.^13,46^ In addition, because female prairie voles are induced ovulators, estrus stage does not need to be quantified when measuring virgin females unexposed to males post weaning.^19^ Despite this, most research on prairie vole communication has focused on vocalizations and not on neural properties of receiving sound information.^9,47^ Mature prairie voles vocalize between 2.5 and 35 kHz, and physics of their middle ear would indicate optimal hearing ranges between 8 and 16 kHz, which is somewhat consistent with our findings of hearing between 8 and 32 kHz, particularly when accounting for higher frequency pup vocalizations.^8,47^ Pup vocalizations are also comparatively “low” with maximal spectra between 22 and 50 kHz, consistent with the broad hearing range.^40^ The fact that there are few differences between males and females in ABR-measured hearing range may well be consistent with the presence of strong biparental care given that males and females respond similarly to pup ultrasonic vocalizations.^2,3^ It would be interesting to directly compare similar measures and potential sex differences to other species with similar parental care and breeding strategies such as Mongolian gerbils and guinea pigs.^12,17^ These results are also consistent with broad tuning of auditory responses found in other rodents, such as *Onychomys leucogaster* (northern grasshopper mice, broad sensitivity from 8 to 24 kHz),^48^ which are consistent with the matched filter hypothesis that proposes that sensory systems evolve to detect the most ecologically relevant stimuli.^49^

Male prairie voles did weigh significantly more than females; however, increased mass does not translate to overall larger heads or pinna. Rodents have been postulated to hear high frequencies due to the limitations of their small head sizes to create ILD cues.^50^ However, rodents also have some of the broadest hearing ranges of any mammal.^18^ Important aspects of hearing ability and sound localization are the size and shape of the head and pinna of an animal. Pinnae have an important role in vertical sound localization and removal of front-back confusion.^51^ Although head size is known to be positively correlated with body size across taxa, general size differences between male and female prairie voles appear to be negligible regarding pinna and head size. Many species have notable secondary peaks of high frequency hearing, and prairie voles are potentially no exception with a secondary peak around 32 kHz, although note that closed sound systems are difficult to calibrate at high frequencies. These secondary peaks can be attributed to pinna size, shape, and directionality, and the smaller the size of the head, typically, the higher frequency hearing^52^ with notable exceptions for some taxa. Our HRTF results suggest prairie voles have ITD sensitivity consistent with their head and pinna sizes (∼± 80 *μ*s, calculated as pinna width plus inter pinna distance divided by the speed of sound, 343 m/s). The general slope and range of cues match well with the expected ranges based on their head features and are consistent with similar measurements in other species (human,^34,53,54^ cat,^55–57^ monkey,^58,59^ ferret,^60,61^ tammar wallaby,^62^ various species of bats,^63–66^ gerbil,^41^ chinchilla,^67,68^ mouse,^69,70^ rat,^71,72^ rabbit,^73^ barn owl,^74,75^ guinea pig,^33^ and frilled neck lizard,^35^ among others). Unsurprisingly, as pinna and head size were similar between the sexes, HRTF measurements did not show clear differences between the sexes.

It is possible that prairie voles can hear even higher than the measured 46 kHz or specific directional information is missing in these measurements, such as spectral notches, that would appear in higher frequencies than those presented for our HRTF measurements (limited to <20 kHz). The use of quantitative methods for analyzing threshold of hearing responses may have been beneficial in this study, however, due to time limitations of anesthesia and length of BIC recordings, threshold measurements were made in real-time with fewer repetitions when clear ABR waveforms were present at higher intensities, allowing for quicker but more subjective threshold measurements. Previous work has also shown that using multiple observers for noisy tone-pip generated ABR waves can be just as effective as more quantitative measures.^16^ In addition, we did not measure enough frequencies in the low hearing range to test the true lower limit of their low frequency hearing, although thresholds of 46–50 dB SPL at 1 kHz suggest good low frequency hearing and access to the ITD localization cue. These thresholds are higher than some low frequency specialists, such as Mongolian gerbils, but differences in setup and anesthesia can make overall threshold comparisons difficult between studies.^76^ The presence of many cells comprising the medial superior olive (MSO), an important structure for mammalian low frequency hearing, would indicate that they likely have low frequency hearing as well, making them more similar in hearing range to the Mongolian gerbil (*M. unguiculatus*) than the house mouse (*Mus musculus*).^1^

## V. CONCLUSIONS

This is the first study to measure the hearing ability of prairie voles, *M. ochrogaster*, including morphology, and monaural and binaural responses. Importantly, we measured whether there were sex differences in hearing range, morphology, and monaural or binaural hearing. We predicted, due to the biparental care, similarity in head size and monogamy in this species, and that there would be limited sex differences in hearing ability. We showed that although there were no differences in the sexes for morphological measures, HRTFs, hearing range (tone-evoked ABRs), and binaural measurements, there were significant differences in click-evoked monaural amplitudes of peripheral auditory brainstem waves (I and II) and increased latency in waves III and IV of females. These findings are significant because they indicate that in some species, sex differences may be limited to certain aspects of hearing, such as the periphery. The mechanisms that underly these differences in amplitude and latency of monaural responses are an intriguing area for future research and may provide insights into hearing differences observed in other species.
